# Supplementary material for: Learning curve and surgical outcome of robotic assisted colorectal surgery with ERAS program
Source: Sci Rep. 2022 Nov 29;12:20566. doi: 10.1038/s41598-022-24665-w (PMC9709162; doi:10.1038/s41598-022-24665-w)
Supplement: Supplementary file 1 — Supplementary Information 1. [file 41598_2022_24665_MOESM1_ESM.zip › LSC Raw data-Ñ[▒K/087/P_ñw╝╨░O▒Kñσ.pdf]

[redacted]

Pathologic diagnosis:
Colon, terminal ileum, and appendix, right hemicolectomy ---
Tubulovillous adenoma with focal high-grade dysplasia. The appendix, ileocecal valve, bilateral cut ends and mesocolic lymph nodes (0/31) are free of tumor.

Ancillary study for diagnosis:
Step cut x 3 done.

Gross description:
The specimen consists of a segment of colon, 17 cm in length, and 8 cm in proximal circumference, 6 cm in distal cut end circumference, and a segment of terminal ileum, 9 cm in length, and 3 cm in cut end circumference. An unremarkable appendix, 5 cm, is noted. A gray tan soft cauliflower-like tumor, 2.5x2x3 cm, is noted, 8 cm to the distal cut end. On cross section, the tumor confined to the mucosa with 10 cm to the radial margin grossly. The appendix, ileocecal valve, and both cut ends are free of tumor. Representative part for section: A) proximal cut end B) distal cut end C) ileocecal valve D-F) tumor G) appendix H) vascular cut end J-L) lymph nodes.

Microscopic description:
Sections show colon mucosa with tubulovillous proliferation with dysplasia. Focal high-grade dysplasia is found.

Comment:
Further clinical follow-up is suggested.
#T-59420_2 #M-82630_2 0 1105 000000
#T-59420_2 #M-74003_2 0 1105 000000
#T-C4560_2 #M-09183_2 0 1105 000000
#T-59200_2 #M-09183_2 0 1105 000000

已電子簽章

敬告：本單僅供醫師參考使用，不具任何申請效力！！
請病患妥為保管，若不慎遺失需自負個人資料外洩責任！
